# Correction to: Development and Validation of a Nonremission Risk Prediction Model in First-Episode Psychosis: An Analysis of 2 Longitudinal Studies

**DOI:** 10.1093/schizbullopen/sgad007

**Published:** 2023-04-03

This is a correction to: Samuel P Leighton, Rajeev Krishnadas, Rachel Upthegrove, Steven Marwaha, Ewout W Steyerberg, Georgios V Gkoutos, Matthew R Broome, Peter F Liddle, Linda Everard, Swaran P Singh, Nicholas Freemantle, David Fowler, Peter B Jones, Vimal Sharma, Robin Murray, Til Wykes, Richard J Drake, Iain Buchan, Simon Rogers, Jonathan Cavanagh, Shon W Lewis, Max Birchwood, Pavan K Mallikarjun, Development and Validation of a Nonremission Risk Prediction Model in First-Episode Psychosis: An Analysis of 2 Longitudinal Studies, *Schizophrenia Bulletin Open*, Volume 2, Issue 1, January 2021, sgab041, https://doi.org/10.1093/schizbullopen/sgab041

During the original analysis, we combined the data across the 10 multiple imputations into a single dataset from which we determined the C-statistic performance, and calibration intercept and slope. This resulted in estimates of the values with narrower confidence intervals than if we had correctly applied Rubin’s Rules.(1)

In addition, we standardised (centred and scaled) the development (NEDEN) and validation cohort (Outlook) data separately. This is not best practice and instead we should have used the means and standard deviations from the development cohort to standardise the validation cohort.(2, 3)

The revised Table 1 shows the final logistic regression nonremission prediction model specification. We now provide mean and standard deviation values from the development cohort to allow the transformation of the predictor variables to Z-scores for their use in the model. This was omitted from the original published paper but is required to apply the model to new patients.

**Table 1.** The final logistic regression nonremission prediction model specification. We now provide mean and standard deviation values to allow the transformation of the predictor variables to Z-scores for their use in the model. This was omitted from the original published paper but is required to apply the model to new patients.

Variable	Values to transform to Z-score	Unadjusted Final Model		Adjusted Final Model (Shrinkage Factor = 0.84)	
	Mean (SD)	β Coefficient (95% CI)	Odds Ratio (95% CI)	β Coefficient	Odds Ratio
Intercept		0.022 (-0.334, 0.379)		0.029	
Male Sex (1 or 0)	N/A	0.259 (-0.129, 0.646)	1.295 (0.879, 1.908)	0.217	1.242
Age at Study Entry	22.51 (4.887)	-0.037 (-0.210, 0.137)	0.964 (0.810, 1.147)	-0.031	0.970
Past Drug Use (1 or 0)	N/A	-0.101 (-0.478, 0.277)	0.904 (0.620, 1.319)	-0.084	0.919
DUP (days)	307.5 (632.3)	0.546 (0.255, 0.838)	1.727 (1.291, 2.311)	0.460	1.581
PAS Highest Functioning Achieved	1.745 (1.446)	0.427 (0.241, 0.613)	1.533 (1.273, 1.847)	0.358	1.431
PANSS P1 Delusions	2.828 (1.683)	0.060 (-0.166, 0.287)	1.062 (0.847, 1.332)	0.051	1.052
PANSS P2 Conceptual Disorganization	1.945 (1.254)	-0.359 (-0.568, -0.151)	0.698 (0.567, 0.860)	-0.301	0.740
PANSS P3 Hallucinatory Behavior	2.931 (1.686)	0.543 (0.334, 0.753)	1.722 (1.396, 2.123)	0.455	1.577
PANSS N4 Passive Social Withdrawal	2.68 (1.576)	0.346 (0.146, 0.545)	1.413 (1.157, 1.725)	0.290	1.336
PANSS G6 Depression	3.229 (1.681)	-0.198 (-0.398, 0.002)	0.820 (0.672, 1.002)	-0.166	0.847
Insight Scale – Nervous or Mental Illness	1.288 (0.7951)	-0.075 (-0.263, 0.114)	0.928 (0.768, 1.121)	-0.062	0.940
GAF Symptoms	51.48 (16.72)	-0.272 (-0.540, -0.005)	0.762 (0.583, 0.995)	-0.228	0.780
GAF Disability	53.27 (15.58)	-0.019 (-0.267, 0.229)	0.981 (0.765, 1.257)	-0.016	0.984
Average Deprivation Score in Patient’s PCT	27.27 (12.22)	0.221 (0.029, 0.414)	1.248 (1.029, 1.513)	0.185	1.204

DUP = duration of untreated psychosis; PAS = premorbid adjustment scale; PANSS = Positive and Negative Syndrome Scale; GAF = Global Assessment of Functioning; PCT = Primary Care Trust; N/A = not applicable

The correct confidence intervals for the internal validation C-statistic and calibration slope are 0.74 (0.72, 0.76) and 0.84 (0.76, 0.92), respectively.

The correct values for the external validation C-statistic, calibration intercept and slope are 0.73 (0.64, 0.81), -0.014 (-0.34, 0.31) and 0.85 (0.42, 1.27). The revised Figure 2 shows the external validation calibration plot.

**Figure 2:**
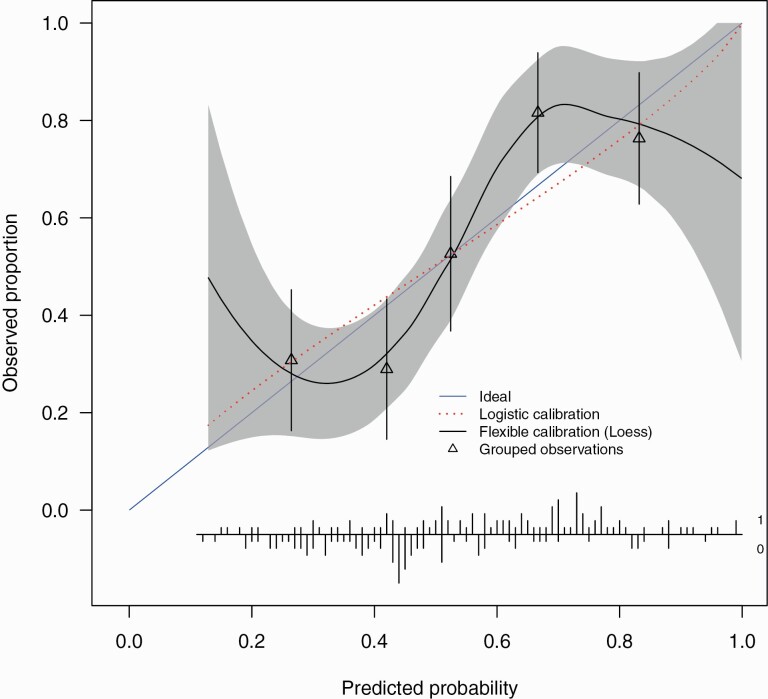
External validation calibration plot. The calibration intercept of -0.014 (-0.34, 0.31) and slope 0.85 (0.42, 1.27). Triangles represent quintiles of subjects grouped by similar predicted risk. The distribution of subjects is indicated with spikes at the bottom of the graph, stratified by endpoint (nonremitters above the x-axis, remitters below the x-axis). Although both sets of confidence intervals overlapped the ideal values, the calibration slope point estimate is smaller than 1 indicating that the predicted risks were too extreme in the sense of overestimating for patients at high risk while underestimating for patients at low risk and is indicative of overfitting of the model. The calibration intercept point estimate was close to ideal suggesting no general over- or underestimation of predicted risks.

In terms of the external validation net-benefit, the revised Figure 3 shows that between thresholds of 35% to 70% treating based on our model is better than treating all, treating none or treating using DUP alone. At a probability threshold of 50% (midpoint of the range of clinician chosen thresholds), treating based on our model has an increased net-benefit of 16% compared the strategy of treating all.

**Figure 3:**
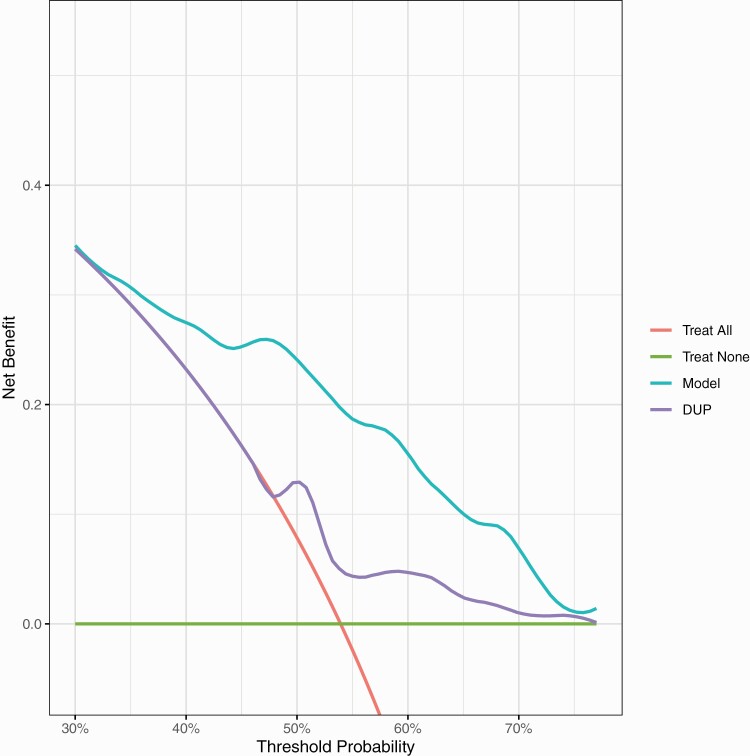
External validation decision curve analysis plot. Net-benefit is the treatment threshold weighted sum of true- minus false-positive classifications for each strategy plotted against an entire range of treatment thresholds. Green line: no patients are treated, net-benefit is zero (no true-positive and no false-positive classifications); red line: all patients are treated; purple and cyan lines: patients are treated if predictions exceed a threshold, with nonremission predictions based on adjusted DUP only, or on our prediction model. Between thresholds of 35% to 70%, treating based on our model is better than treating all, treating none or treating using DUP alone.

Altogether, these results are very similar to the original published results and the interpretation is largely unchanged. The only change is that the external validation calibration slope, although its confidence intervals still overlap the ideal, does suggest a degree of overfitting. The original and updated R code are available online (https://github.com/samleighton87/NEDEN_Outlook_FEP).
